# Counseling supervision for genetic counselors: A proposed outsider witness structure

**DOI:** 10.1002/jgc4.70056

**Published:** 2025-05-14

**Authors:** Mariangels Ferrer‐Duch, Fiona Ulph, Elisabet Dachs Cabanas, Glenda Fredman, Rhona MacLeod

**Affiliations:** ^1^ Riverbank Psychology Manchester UK; ^2^ Cures Integrals i Serveis de Salut, Universitat de Vic, Universitat Central de Catalunya Vic Spain; ^3^ Division of Evolution, Infection and Genomics, School of Biological Sciences University of Manchester Manchester UK; ^4^ Manchester Centre for Health Psychology, Division of Psychology and Mental Health, School of Health Sciences, Faculty of Biology, Medicine, and Health University of Manchester Manchester UK; ^5^ Psychology Programme University of Hertfordshire Hertfordshire UK; ^6^ Manchester Centre for Genomic Medicine, St Mary's Hospital Manchester University NHS Trust Manchester UK

**Keywords:** counseling supervision, genetic counselor, narrative therapy, outsider witness, structure

## Abstract

Counseling supervision for genetic counselors is recognized as an important aspect of professional registration. Professional bodies in countries, including the United States, Australia, and the United Kingdom, have published recommendations for the delivery of counseling supervision covering such things as access and frequency of delivery. Yet, there has been little written about the theoretical frameworks underpinning counseling supervision or how these have been applied within this setting. We present a structure for group counseling supervision for genetic counselors (GCs), informed by narrative therapy, and suggest how it can be adapted for online delivery. The format of the group session is detailed to allow for the outsider witness approach to supervision to be replicated in other genetic centers. We wish to encourage more research in this area to explore models of supervision, including involvement of counseling supervisors trained in strength‐based approaches such as narrative and compassion‐focused therapy. These types of counseling approaches to supervision may be beneficial for sustaining staff morale and team working, particularly important at a time when demands on genetic services are increasing. Effective models of counseling supervision will help to sustain the work of genetic counselors, which in turn will benefit patients and their families.

**What is known about this topic**

There is little information about counseling supervision and narrative therapy.

**What this paper adds to the topic**

This paper outlines the internal structure of group counseling supervision for genetic counselors, drawing on principles from narrative therapy. It also suggests ways to adapt this approach for online delivery.

## 1 INTRODUCTION

Counseling supervision is recognized as essential for genetic counselors' professional development, well‐being, and teamwork as well as a reflective means of maintaining safe and effective practice (McEwen et al., 2025; Paneque et al., 2023). The Working Group on Genetic Counseling Supervision of the UK and Eire (Ireland) Association of Genetic Nurses and Counselors (AGNC) defined counseling supervision as follows:

Genetic counseling supervision is a formal and contractual arrangement, whereby genetics meet with a suitably trained and experienced supervisor to engage in purposeful, guided reflection of their work. Focusing on the dynamics between client and genetic counselor, the aim of this process is to explore the interaction between the counselor and their client, and the impact of external factors on this, enabling counselors to learn from experience, improve their practice and maintain competence. The overall intention is to enhance the quality and safety of client care and to promote the ongoing professional development of the genetic counselor. (Report from the UK and Eire Association of Genetic Nurses and Counsellors (AGNC) Supervision Working Group on genetic counselling supervision, 2022)

A clear distinction is made between clinical supervision provided to trainee genetic counselors and counseling supervision (Clarke et al., 2007). In countries where the profession is recognized and regulated (whether voluntary or statutory registration), most GCs will have access to either individual or group counseling supervision (McEwen et al., 2025). The provision of counseling supervision overall is, however, very variable across most of Europe (Guimarães et al., 2024; Paneque et al., 2023). Internationally, the hope is for greater government recognition of the profession of genetic counselors, where this is not yet the case (Ormond et al., 2024). In turn, this will help to support GC professional bodies in making a case for counseling supervision where none already exists.

In countries where the profession and counseling supervision are well established, for example the United States, United Kingdom, and Australia, policy documents are regularly updated outlining supervision requirements for professional registration (McEwen et al., 2025).

The extent to which a particular counseling model underpins counseling supervision is largely informed by the counseling supervisor and their particular training and background (Middleton et al., 2007). There has been little research to date investigating this more fully, including how the models utilized in counseling supervision may influence the genetic counselors' practice (Clarke et al., 2007). While international requirements may vary, any model of counseling supervision should be able to be adapted both to the professional recommendations of the country, as well as to the needs of the group.

This article will focus on a particular approach to genetic counselor group supervision that has evolved in Manchester, UK over the last 10 years. The groups include both registered and trainee genetic counselors with a range of years of experience and areas of clinical expertise. The approach is based on the narrative model (White & Epston, 1990a). The narrative therapy model is dedicated to understanding and addressing the inherent complexities of human narratives and processes of personal transformation (Denborough, 2019; Gonçalves et al., 2009; White & Epston, 1990b). Given that the work of genetic counselors involves navigating multiple layers of complexity, including both medical and psychosocial aspects, the narrative approach holds significant potential for providing support in these contexts.

Originally, the supervision was conducted in person, but over the past 5 years, it has transitioned to an online format which helps to support hybrid ways of working for genetic counselors. Each counseling supervision group comprises 6–8 genetic counselors and meets monthly for 2 h of online counseling supervision.

## 2 NARRATIVE THERAPY

The Narrative approach was developed by Michael White and David Epston over many years of working with families in the contexts of family therapy and social work (White, 2007; White & Epston, 1990a). This approach is vitally interested in the stories of people's lives and how stories can be told in ways that make people stronger (Morgan, 2000; White, 2007; Wingard & Lester, 2001). The theoretical influence of the narrative model encompasses social justice theory, anthropology, and feminism (Freedman & Combs, 1996; Russell & Carey, 2003; White & Epston, 1990a).

## 3 A NARRATIVE INFORMED SUPERVISION APPROACH

**FIGURE 1:**
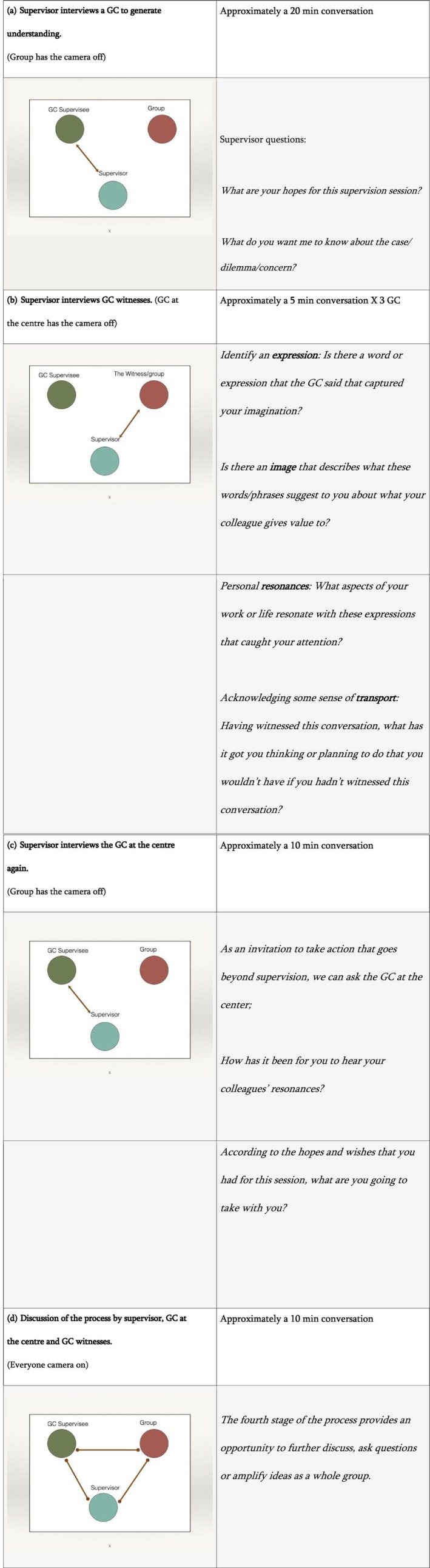
Outsider witness (OW). Adapted from the work of Michael White (2007, pp. 190–192).

A narrative supervisor will aim to align supervisory practices with the principles and social values of narrative therapy (Whiting, 2007). It seeks to have a collaborative approach (White, 1997), staying curious and seeing supervises with skills and abilities to resolve their own dilemmas (Kahn & Monk, 2017; Whiting, 2007). The narrative supervisor aims to strengthen and richly describe the knowledge (White & Epston, 1990a) of the supervisees in ways that provide them with “new options for action in addressing their concerns” (White, 1997, p. 56). The types of narrative style questions that the supervisor may use during supervision are presented in Figure 1.

## 4 THEORETICAL APPROACH TO SUPERVISION

The proposed approach to supervision includes outsider witness (OW) practices that draws on the work of the anthropologist Barbara Myerhoff (Carey & Russell, 2003; Myerhoff, 1986; White, 1997) and is influenced by the family therapy practice of “reflecting teams” (Andersen, 1987). Whilst the format for supervision is similar whether delivered in person or online, we will highlight here how we have adapted the process effectively for online groups.

## 5 SUPERVISION STRUCTURE AND APPROACH

At the start of supervision, there is time for reflection and sharing from the last supervision session or anything that has arisen in the interim, e.g. a concern outside the supervision space. Everyone has their cameras on and actively participates at the beginning of the session. The GC participants then negotiate who will present a case or a dilemma to supervision. The decision is influenced by what feels most urgent or important, depending on discussions within the group. When genetic counselor supervision groups are well established, this process becomes an opportunity to practice connection and solidarity between the GCs.

The supervision session then uses the OW approach that is structured in four stages (see Figure 1); identification of an expression, explaining an image that represents that expression, describing personal resonances, and acknowledging transport. It focuses on one member of the supervision group (the GC at the center), discussing with the supervisor a dilemma or case (Figure 1a). Both the GC at the center and the supervisor have their cameras on, while everyone else has theirs off. This creates an intimacy that facilitates meaningful conversations with the supervisor. The rest of the GCs listen and three of them respond as outsider witnesses (Fox & Tench, 2002; Nadan, 2020; White, 2007, pp. 185–218). This witnessing structure (Freedman, 2014) enables the GC at the center to share what is relevant to them and allows the outsider GCs to amplify and validate the narrative that they witness (Carey & Russell, 2003; Nadan, 2020; Walther & Fox, 2012).

The supervisor prepares the GC outsider witnesses by encouraging them to take notes and listen in a particular way (Figure 1b). The supervisor then interviews each of the GC outsider witnesses using the four stages of this structure: identification of an expression, explaining an image that represents that expression, describing personal resonances, and acknowledging transport (Figure 1b). They are invited to join the supervisor and switch on their cameras when they enter the conversation again creating a more private conversation where it is possible to reflect without being influenced by others in the group. The GC at the center is able to switch off the camera and quietly listen to the responses of each of the outsider witnesses. The supervisor then invites the GC at the center to rejoin for a second conversation (camera on for the supervisor and the GC at the center) and is interviewed as a witness to the responses of the three GC colleagues (Figure 1c). At this point, the GC outsider witnesses have switched off their cameras.

The last step of this structure is the discussion of the process by the supervisor, GC at the center, and GC witnesses (Figure 1d). At this point, everyone has the camera switched on.

In this paper, we adopt a pragmatic approach to describe the bare structure of a narrative counseling supervision group for genetic counselors, aiming to provide clarity regarding the process. A further in‐depth consideration of the principles of narrative theory and how these may be applied in the field of genetic counseling can be found in our earlier paper (MacLeod et al., 2021). The structure of the session can be used to support reflective practice. We have not explored the content or themes that emerge in supervision. This is elaborated more fully in a paper by the first author (Ferrer‐Duch, 2025). The effectiveness of this type of counseling supervision from the perspective of GC attendees is being explored in the context of an ongoing research project. However, online anonymous evaluations of supervision by the GC team in Manchester suggest that the approach is highly valued. Feedback highlights its “energizing” effects, fosters a sense of professional development among GCs, and enhances feelings of connection within the group. Genetic counselors attending the narrative supervision group have spoken of feeling “lighter” after the session, regardless of whether or not they are the GC at the center. Moreover, they share that, as a result of counseling supervision, ideas from narrative practices are being integrated into their work with individuals and families. Interestingly, GCs have consistently advocated for the benefits of having GCs with varying levels of experience within each supervision group.

## 6 CONCLUSION

In this paper, we describe a particular structure for online group counseling supervision for genetic counselors informed by narrative therapy. Our approach to supervision has evolved over a 10‐year period through the close collaboration between the counselor supervisor (MF‐D, the first author) and the GC supervisees. In its early stages, the same structure was implemented in person, providing similar effects. The group would sit in a semicircle, with the supervisor setting their chair to focus on one GC at a time, first on the presenter, followed by each of the other OW.

We have presented the process followed in counseling supervision as a way of making this more visible and to open more conversations in relation to how counseling supervision is delivered. We hope this will encourage more research and similar sharing of supervision formats and theoretical adaptations (Guimarães et al., 2024; McEwen et al., 2025). There have been pleas in the literature for more studies exploring the interior of genetic counseling consultations, the “black box” of the process. (Biesecker & Peters, 2001). McEwen et al. (2025) in an in‐depth overview of genetic counseling supervision, call for an international community of supervision practice as a welcome means of sharing practices and resources. With our paper, we hope to contribute to ideas for sharing practices.

This paper outlines the interior of a specific approach to counseling supervision. To our knowledge, this is the first paper to propose a narrative approach to counseling supervision for genetic counselors. While this approach has not been formally evaluated in the context of genetic counseling supervision, there is evidence of its utility in other settings such as family therapy (Nadan, 2020).

Narrative practices seem to be a promising way of helping people connect and in facilitating family communication about genetic risk information (MacLeod et al., 2021; Spiers et al., 2020; Stopford et al., 2020). More research is needed in relation to how the approach may be effective for genetic counseling practice (Dane et al., 2025; Guerra et al., 2023) and counseling supervision. Qualitative inquiry may be a way of teasing out the nuances of supervision and the perceptions among GCs regarding its impact on their practice. Future research may include innovative qualitative approaches that include co‐design with GC supervisees. Such a method would align well with narrative practices, as it places collaboration at the center and involves the GCs from the outset. This type of participatory approach to research aims to allow people to be involved in the research process, “knowledge is built out of the collective comparison of subjective experiences of reality by groups of people commonly exposed to, acting on and/or with first‐hand experience of that reality” (Loewenson et al., 2014, p. 20).

It is likely that the next decade will see more people coming forward for genomic counseling and testing and changes in the way services are delivered (Do et al., 2024). We can anticipate that counseling supervision will play an even greater role in sustaining genetic counselor wellbeing and in supporting team working. Recent research publications point to the challenges for genetic counselors of burnout and compassion fatigue (Allsbrook et al., 2016; Caleshu et al., 2022). At the same time, there are opportunities for increased team working and expanding our knowledge and understanding of some of the strength‐based approaches that have proven effective in other areas of psychology and healthcare (Davies et al., 2024; Rajaei & Jensen, 2020; Williams‐Reade et al., 2014). Narrative therapy is one such approach, and research is in progress to look at this in more depth in relation to GC counseling supervision. We hope that other counseling supervisors and GC teams will be encouraged to present their methodologies and theoretical frameworks for supervision to enable others to replicate or adapt to their own practice and setting.

## AUTHOR CONTRIBUTIONS

Mariangels Ferrer‐Duch, conceptualisation, writing – original draft. Fiona Ulph, Supervision and editing. Elisabet Dachs Cabanas, review and editing. Glenda Fredman, writing, review and editing. Rhona MacLeod, conceptualisation, writing – original draft.

## FUNDING INFORMATION

The authors received no financial support for the authorship, and/or publication of this article.

## CONFLICT OF INTEREST STATEMENT

The authors declared no potential conflicts of interest regarding authorship, and/or publication of this article.
